# Optimization and Chemical Characterization of Biosurfactant Produced from a Novel *Pseudomonas guguanensis* Strain Iraqi ZG.K.M

**DOI:** 10.1155/2023/1571991

**Published:** 2023-02-01

**Authors:** Zeena Ghazi Faisal, Mayaada Sallal Mahdi, Khalid H. Alobaidi

**Affiliations:** ^1^Department of Biology, College of Education, Al-Iraqia University, Baghdad, Iraq; ^2^Department of Molecular and Medical Biotechnology, College of Biotechnology, Al-Nahrain University, Baghdad, Iraq; ^3^Department of Plant Biotechnology, College of Biotechnology, Al-Nahrain University, Baghdad, Iraq

## Abstract

Microbial surfactants are widely used in medical, pharmaceutical, agricultural, industrial, food, and cosmetics applications. In the present study, 85 indigenous bacteria were isolated from petroleum-contaminated soils of the Al Dourah refinery, electric power station, and electric generators in Baghdad, Iraq. Twenty nine isolates gave positive results in both blood agar and blue agar medium and were secondarily screened. One isolate was selected as a potent biosurfactant producer and molecularly identified and recorded in the NCBI GenBank nucleotide sequence database as *Pseudomonas guguanensis* strain Iraqi ZG.K.M. In optimized conditions, this strain can produce about 3.01 g/l of biosurfactant. The product could reduce the surface tension from 72 to 38 ± 0.33 mN/m and have E_24_% of 52 ± 0.33%. This biosurfactant was preliminarily specified to be a glycolipid and characterized as a rhamnolipid with anionic nature, usually to be a monorhamnolipid as evident from TLC, FTIR, and GC-MS analyses.

## 1. Introduction

Microbial compounds which exhibit a clear surface activity are classified as biosurfactants. They belong to different groups including glycolipids, phospholipids lipopeptides, fatty acids, polysaccharide-protein complexes, and neutral lipids. These molecules can perform different natural roles in the growth and reproduction of microorganisms [1]. Biosurfactants are organic, surface-active amphiphilic compounds that contain both hydrophobic and hydrophilic moieties, produced mainly by different microorganisms (bacteria, yeast, or fungi) on cell surfaces or can be secreted extracellularly [1, 2]. They could accumulate between fluid phases and help in reducing the surface and interfacial tensions, which will make them potential candidates for boost oil recovery, biodegradation, and bioremediation [3–8].

With a worldwide preference for renewable products, attention to microbial surfactants has been increased due to their diversity, selectivity, low toxicity, biodegradability, ecological acceptance, effectiveness at extreme pH and temperatures, the possibility to be produced through fermentation, and widespread applicability [4, 9, 10]. Nowadays, biosurfactants are used in industries such as pharmaceuticals and cosmetics and have emerged as potential agents in food industries and agriculture, possessing several interesting properties of medical importance, bioremediation of petroleum pollutants, management and enhancing crude oil recovery, lubrication, solubilization, wetting, detergency, and recently biosurfactants have been found to disrupt biofilm formation [2–9].

A wide range of bacteria have been reported as biosurfactant producers. The genera *Pseudomonas* and *Bacillus* are extremely studied as rhamnolipids and lipopeptides producers [10–13]. The characteristics of biosurfactants depend not only on the producer's organisms but also on the growth conditions. So, it will be necessary to assess various available strains for their biosurfactant potential, the suitable nutrients, and the cultural conditions required to achieve high productivity [4, 10].

In the present study, a biosurfactant-producing bacterium, *Pseudomonas guguanensis* strain Iraqi ZG.K.M, was isolated from oil-contaminated soils. Moreover, optimization of bacterial growth conditions to enhance the biosurfactant production and characterization of the product for its chemical nature were carried out.

## 2. Materials and Methods

### 2.1. Collection of Soil Samples

Approximately 10 g of soil sample was collected from the topsoil (5–15 cm) of the Al Dourah refinery, electric power station, and electric generators in Baghdad. Soil samples were stored in sterile, well-labeled polyethylene bags that were tightly packed and transferred to the laboratory aseptically and refrigerated at 4°C until use.

### 2.2. Isolation and Purification of Biosurfactant-Producing Bacteria

A selective enrichment method was applied for the isolation of biosurfactant-producing bacteria from samples of petroleum-contaminated soil [14]. Modified mineral salt media (MSM), composed of (g/l) KH_2_PO_4_ (1.0), K_2_HPO_4_ (1.0), NaCl (1.0), CaCl_2_ (0.05), (NH_4_)_2_ SO_4_ (1.0), MgSO_4_.7H_2_O (0.5), FeCl_3_ (0.002), and yeast extract (0.1), was used in this technique [15]. One gram of soil sample was added to 250 ml Erlenmeyer flasks, each containing 100 ml of sterilized MSM with 2% crude oil as the sole carbon source. The flasks were incubated at 35°C in a (150 rpm) shaker incubator for 7 days. After a week of incubation, 1 ml of the first culture was transferred into 100 ml sterilized fresh MSM with 2% crude oil and incubated again under the same conditions for 7 days. This process was repeated three times to decrease the unwanted microbial load. After three cycles of enrichment, 1 ml from each culture broth was used for serial dilutions, starting from 10^−1^ to 10^−5^, and then, 0.1 ml from each dilution was spread on nutrient agar plates that incubated at 35°C for 24 h. Bacterial colonies with different morphologies were selected and purified using the streaking method on nutrient agar plates. The method was repeated until pure colonies were obtained and stored as aliquots in frozen conditions. For each experiment, the aliquots were thawed and used.

### 2.3. Screening for Biosurfactant-Producing Bacteria

To select the most potent biosurfactant producers, the previously isolated bacteria were cultivated in MSM with 2% crude oil and incubated for 7 days at 35°C in a (150 rpm) shaker incubator.

#### 2.3.1. Primary Screening

Eighty-five bacterial isolates were screened by the plate assay, using blood agar, hemolysis test [16], blue agar medium, and CTAB agar test [17]. 50 *μ*l of each bacterial culture was transferred into wells punctured in plates of blood agar and blue agar medium. Plates were then incubated for 48 h at 35°C. The development of a yellow transparent zone or bluish halo zone around wells of blood agar and blue agar medium indicates a positive result of biosurfactants production.

#### 2.3.2. Secondary Screening

Twenty-nine bacterial isolates that gave positive results in both blood agar and blue agar medium were screened to select the most promising biosurfactant-producing isolates. Bacterial isolates were cultivated in MSM containing 2% crude oil and incubated in a 150 rpm shaker incubator at 35°C. After 7 days of incubation, the cultures were centrifuged at 12.000 g for 15 min at 25°C. Cell-free supernatants were screened for the presence of biosurfactant, based on the drop collapse test, oil spreading technique, measurement of surface tension, and emulsification index, while cell pellets were used for measuring cell hydrophobicity by bacterial adherence to hydrocarbons (BATH) assay [2, 10, 14, 16–18].

*(1) Drop Collapse Assay*. A single drop of crude oil was set on a clean glass slide; then, a single drop of cell-free supernatant was dropped onto the crude oil drop, and the drop size was observed 1 min later. When the droplets are in a flat shape, the result was considered positive for biosurfactant production. Distilled water was applied as a negative control [14, 16].

*(2) Oil Spreading Technique*. Briefly, about 20 ml of distilled water was poured into a Petri dish of 90 mm diameter. Then, 1 ml of crude oil was spread on the surface of the water until the formation of a thin oily layer. After that, 500 *μ*l of cell-free supernatant was gently spotted on the center of the oily layer surface. The diameter of the oil spreading area was measured after 30 s. Distilled water was applied as a negative control [14, 16–18].

*(3) Measurement of Surface Tension*. Surface tension is an important parameter for the evaluation of surface activity, determined at room temperature by a K6 tensiometer using the du Nouy platinum ring method. Before measuring, the tensiometer must be calibrated with distilled water (72 mN/m). About 20 ml of cell-free supernatant was transferred into a clean glass beaker and placed on the sample table. The height of the sample pool was adjusted, so that the platinum ring, hanging from the balance hook, was immersed under the liquid surface of the sample for 15 min to be equilibrated and then carefully pulled up. The microbalance records the force applied to the ring while pulling through the liquid surface. When the platinum ring leaves the liquid level, the value is displayed as the surface tension of that sample. The noninoculated medium was used as a control [2, 16, 18].

*(4) Measurement of Emulsification Index (E_24_)*. Equal volumes (v/v) of cell-free supernatant and toluene were injected into a 10 ml test tube and mixed vigorously by vortex for 2 min. Test tubes were placed vertically at room temperature for 24 h without disturbance. The height of the emulsifier layer was measured, and E_24_ was calculated as a percentage of the height of the emulsifying layer (mm) to the total height of the liquid column (mm) multiplied by 100 [16].

*(5) Bacterial Adhesion to Hydrocarbons (BATH) Assay*. The hydrophobicity of the bacterial cells was measured by BATH assay [10]. First, the cell pellet was washed twice with phosphate buffer salt solution (g/l, 16.9 K_2_HPO_4_, and 7.3 KH_2_PO_4_) and then suspended with the same buffer solution to an optical density (OD) of about 0.5 at 600 nm. In a test tube, 100 *μ*l of crude oil was added to 2 ml of cell suspension and the vortex shook for 3 min. After mixing, the aqueous phase and crude oil were allowed to be separated for 1 h. OD of the aqueous phase was then measured at 600 nm. From the OD values, the percentage of cells adherence to the crude oil was calculated using the following formula:

(1) $$\begin{array}{c} \%\;of\;bacterial\;cell\;adherence=\left(1-\left(\frac{ODshaken\;with\;oil}{ODoriginal}\right)\right)\times 100\text{.} \end{array}$$

### 2.4. Identification of the Most Potent Bacterial Isolates

The potent biosurfactant-producing bacterial isolate was studied for its morphological and culture characteristics on a nutrient agar plate. The identification of the isolate was carried out by 16S rRNA sequencing using universal primers, 27F: 5′-AGAGTTTGATCCTGGCTCAG-3′ and 1492R: 5′TACGGTTACCTTGTTACGACTT-3′. The taxonomic analysis and phylogenic tree were prepared by BLAST with the database of NCBI GenBank.

### 2.5. Optimization of Biosurfactant Production by the Identified Strain

To obtain high productivity of biosurfactant, growth optimization was performed to select the critical medium components and optimum cultivation conditions by changing one variable at a time, keeping other factors fixed at a specific set of conditions. First of all, inoculum of the identified strain was prepared by transferring one colony of fresh bacterial culture to a 100 ml Erlenmeyer flask containing 50 ml of sterilized nutrient broth medium. Flask has been incubated overnight in a (150 rpm) shaker incubator at 35°C. This inoculum was used in optimization experiments of biosurfactant production by the identified strain. Factors considered in these experiments are shown in Table 1. All of the experiments were conducted in a 250 ml Erlenmeyer flask containing 100 ml of sterilized MSM. Surface tension and emulsification activity were determined as a response [2, 15].

### 2.6. Production and Extraction of Biosurfactant

Crude biosurfactant was obtained by the acid precipitation and solvent extraction method, using ethyl acetate or chloroform : methanol (2 : 1 v/v) [2, 19, 20]. A fresh culture of the selected strain (1 ml) was inoculated into a flask containing 1 L of mineral salt medium (pH 7) containing sesame oil (4%), NH₄NO_3_ (1%), and salt (1%). Flask was incubated at 30°C in a shaker incubator (150 rpm) for 4 days. After an incubation period, the supernatant of bacterial culture was obtained by centrifugation at 12.0000 g for 15 min at 25°C. The clear supernatant served as the source of crude biosurfactant. The pH of the collected supernatant was adjusted to 2 and kept at 4°C overnight. Equal volume of ethyl acetate or chloroform : methanol (2 : 1 v/v) was added at room temperature and mixed well to ensure suitable mixing. White-colored precipitate indicates the presence of biosurfactant. The organic phase (extract) was separated from the aqueous phase (solvent) by a separation funnel. The emulsifier layer was collected in a glass Petri dish and dried at 40–45°C. A dark yellowish oily precipitate was recovered as a crude biosurfactant. The dry weights were determined and then stored at 4°C for further studies.

### 2.7. Characterization of Partially Purified Biosurfactant

#### 2.7.1. Determination of Ionic Nature

The agar double diffusion method was used to determine the ionic charge of partially purified biosurfactants. Briefly, three uniformly spaced wells were made in a semisolid agar plate (nutrient broth containing 1% agar-agar, poured into a sterile Petri dish). The central well was filled with 50 *μ*l of bacterial culture supernatant. On either side of the middle well, it was filled with a cationic compound (cetyltrimethylammonium bromide (CTAB), 20 mМ) and anionic compound (sodium dodecyl sulfate (SDS), 20 mМ). The plate was incubated at room temperature and monitored over a 48 h period. The ionic nature of biosurfactant was determined by the appearance of a precipitation line between the wells [21].

#### 2.7.2. Thin-Layer Chromatography (TLC) Analysis

A sample (0.1 g) of partially purified biosurfactant was dissolved in methanol at the concentration of 100 *μ*g/ml, then pulled by glass capillary and spotted onto a silica gel plate, prepared in advance, around 1 cm from the bottom. The plate was then placed in a tank containing a mixture of chloroform : methanol : acetic acid (65 : 15 : 2, v/v/v), which served as the mobile phase. The process is complete when the solvent reaches the top of the plate. The silica gel plate was removed and dried in a stream of air. The retardation factor (R_f_) value of biosurfactant bands was calculated as the distance traveled by the samples over the distance traveled by the solvent [17, 22]. The main components of partially purified biosurfactant were identified by the exposure to iodine vapor, or by spraying Molisch's reagent (3.75 g *α*-naphthol in 25 ml of 95% ethanol) and 1% ninhydrin solution (1 g ninhydrin in 100 ml distilled water), for detection of lipids, sugars, and free amino groups, respectively. The plates were then incubated at 100°C for 15 min until the appearance of definite spots [17].

#### 2.7.3. Fourier Transform Infra-Red (FT-IR) Analysis

The functional groups of the extracted biosurfactant were determined qualitatively by FT-IR spectroscopy, based on the oscillation patterns of chemical bonds at characteristic frequencies ranging from 500 to 4000 wave numbers/cm with a resolution of 4 cm^−1^. Extracted biosurfactant sample (0.3–0.5 mg) was placed in spectral grade KBr and processed with IR analytical software [17, 22].

#### 2.7.4. Gas Chromatography-Mass Spectrometry (GC-MS) Analysis

GC-MS analysis was used to identify the number and types of components present in the extracted biosurfactant and their molecular weight. The instrument is equipped with a capillary column ZB-5MS (30 mm × 0.25 mm, I.D. 0.25 *μ*m), using helium (He) as carrier gas with a flow rate of 2 ml/min. The temperatures of the injector and detector were 230°C and 280°C, respectively. A sample (0.1 g) of partially purified biosurfactant was dissolved in methanol at a concentration of 100 *μ*g/ml before being analyzed by GC-MS. The column temperature was initially held at 80°C for 3 min and then increased to 280°C at a rate of 8°C/min and kept for 10 min. A 1 *μ*l sample was then used with a split ratio of 10 : 1 [23].

### 2.8. Data Analysis

All experiments were carried out in triplicate and data were expressed as mean ± SEM. To evaluate statistical significance, one-way analysis of variance (ANOVA) was used for more than two groups. Statistical significance was accepted at *P* < 0.05.

## 3. Results and Discussion

### 3.1. Isolation and Purification of Biosurfactant-Producing Bacteria

Eighty-five different biosurfactant-producing bacteria were isolated by a selective enrichment technique. The process was performed at multiple cycles to ensure bacterial isolates that were obtained at the end of the enrichment cycle could use petroleum compounds rather than to tolerate them. This indicates the ability to collect a large number of bacterial isolates, which are capable of living and utilizing petroleum hydrocarbons, from petroleum-contaminated soil samples. However, there were differences in the number of bacterial isolates in the sampling sites. A possible explanation for this might be associated with the diversity of bacteria capable of degrading hydrocarbons and their derivatives. Additionally, a longer contamination time could lead to be a greater number of microorganisms [24].

### 3.2. Screening for Biosurfactant-Producing Bacteria

#### 3.2.1. Primary Screening

**Figure 1:**
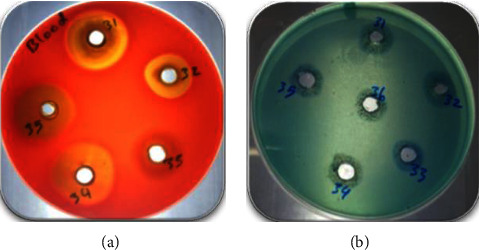
Primary screening of bacterial isolates. *β*-Hemolysis of blood agar medium (a) and bluish halo zone of blue agar medium (b) were considered positive results.

The ability of bacterial isolates to produce biosurfactants was tested by subjecting them to sensitive, easy to use, qualitative assays. These assays include hemolysis and CTAB tests, which can be used only during the isolation stage of biosurfactant producers [12]. Among the 85 examined bacterial isolates, 29 isolates (34%) gave positive results as yellow transparent zones around wells of blood agar medium and bluish halo zone around wells of blue agar medium (Figure 1). These isolates were collected to be secondarily screened for more detection and careful selection of the most promising biosurfactant producers.

#### 3.2.2. Secondary Screening

**Figure 2:**
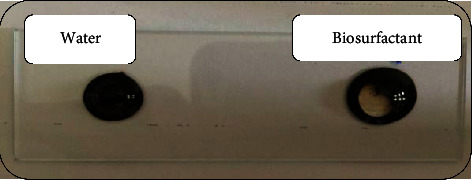
Drop collapse assay. Biosurfactant collapsed the hydrocarbon drop, while water was repelled from the hydrophobic surface.

*(1) Drop Collapse Assay*. It is a qualitative test based on the destabilization of liquid droplets by surfactants. For the 29 bacterial isolates, all gave positive results as the drop spread or even collapse, due to lowering the interfacial tension between the liquid drop and the hydrophobic surface. While, molecules of polar water were repelled from the hydrophobic surface and remained stable, depicted in Figure 2. The stability of the drop depends on surfactant concentration and correlates with surface and interfacial tension [17]. Sidkey and his colleagues recommended the use of both CTAB and dropping collapse tests during the isolation stage of biosurfactant producers because more than one method should be included to identify potential biosurfactant producers. In addition, most of the biosurfactant producers gave positive results in these tests, suggesting that strains highly active at one method were active at the other [12].

**Figure 3:**
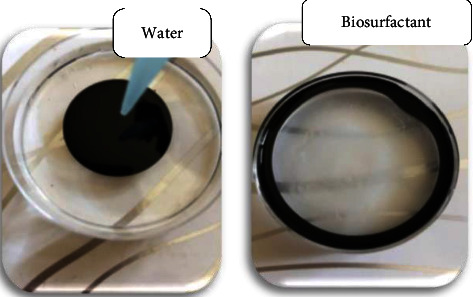
Oil spreading assay technique showing results of the most promising biosurfactant producers. The oil layer was displaced by biosurfactant. Water was a negative control.

*(2) Oil Spreading Technique*. It is quantitative, reliable, rapid, easy to carry out test, and it is used to detect biosurfactant-producing microorganisms. It measures the surface activity of surfactant solution against crude oil. A larger diameter represents a higher activity and higher concentration of the test solution [4, 17, 19]. Results showed different surface activities with a diameter of displaced circle ranging from 3.66 ± 1.33 mm to 77.66 ± 0.33 mm, and no clear zone was observed with water (Figure 3). No relation was found between the drop collapse assay and the oil spreading test, as some isolates found positive with the drop collapse were negative for the oil spreading test. Clearly, Nayarisseri and his colleagues reported the existence of a relationship between drop collapse and oil spreading assays in a way that samples found positive with drop collapse assay were positive for oil spreading test as well [10]. While, Sun and his colleagues showed no relation between these assays, in which some organisms found positive with the drop collapse test were negative for oil spreading and vice versa [17].

*(3) Measurement of Surface Tension*. The tensiometeric technique is a quantitative assay used to select biosurfactant producers by evaluating surface activity through the ability to reduce the surface tension below 72 mN/m [9]. Direct measurement of the surface activity of the culture supernatant is the most straightforward screening method, which gives a strong indication of biosurfactant production [2]. Results showed a reduction in surface tension ranging between 69.33 ± 0.33 mN/m and 44 mN/m, confirming the ability of isolates to produce biosurfactant extracellularly in the culture medium. A direct relation was recorded between drop collapse, oil spreading, and surface tension assays, in which isolates that were highly active in any one of these methods were active in the other two methods. A similar correlation was reported by Nayarisseri and his colleagues [10]. Sidkey and his colleagues stated that biosurfactants produced by *P. aeruginosa* can reduce the surface tension of distilled water from 72 to 30 mN/m [12]. Moreover, biosurfactant extracted from *Enterococcus faecium* MRTL9 reduced the surface tension from 72 to 40 mN/m [25].

*(4) Measurement of Emulsification Index (E_24_%)*. It is an indirect method used for screening biosurfactant production. It is considered a simple, reliable, and rapid test that measures biosurfactant quantity [10]. Results showed a variable emulsification ability ranging from 9.66 ± 0.66% to 64.66 ± 0.33%, indicating the ability of bacterial isolates to produce different amounts of biosurfactants that enhance oil contact with water. In addition, a relationship between E_24_% and surface tension has been demonstrated, where bacterial isolates have been described as being able to produce a good amount of biosurfactant when the surface tension is low and E_24_% is high. Sun and his colleagues found that biosurfactant produced by *P. aeruginosa* has an E_24_% reach up to 61.5% [17]. Ibrahim reported that the cell-free broth of *Ochrobactrum anthropi* HM-1 and *Citrobacter freundii* HM-2 strains successfully emulsified crude oil, diesel, engine oil, sunflower oil, and olive oil and failed to emulsify kerosene and hexadecane [21]. The highest E_24_% value was 90% recorded by *O. anthropi* HM-1 biosurfactant, compared with 89% for *C. freundii* HM-2. In addition, the E_24_% of biosurfactant produced by *Enterococcus faecium* against kerosene oil has been reported to be 64% [25].

*(5) Bacterial Adhesion to Hydrocarbons (BATH) Assay*. BATH assay is an indirect method used to examine the ability of bacteria to produce biosurfactants. The production of surface active compounds makes the surface of cells hydrophobic, which enhances their attachment to the large oil droplets. Therefore, isolates with high hydrophobicity are likely to be more efficient degraders [10]. The results showed a variable ability of bacterial isolates to attach oil droplets ranging from 5.66 ± 0.66% to 88.33 ± 2.18%, which indicates the ability to produce different amounts of biosurfactants. Thavasi and his colleagues reported that strains of the genus *Pseudomonas* exhibited higher adhesion to crude oil than other strains, and the maximum cell attachment to crude oil was recorded with *P. aeruginosa* (95.15 ± 0.21%), making it a more potent biosurfactant producers [26]. On the other hand, Nayarisseri and his colleagues indicated that the highest cell adhesion was observed with *Bacillus* (94.23 ± 0.71%), while *E. coli* showed the lowest adhesion (60.15 ± 1.42%) [9].

From the results of secondary screening, it was seen that bacterial isolates were able to produce different amounts of biosurfactants as compared to each other (Table 2). Isolates possessing high E_24_%, flat drops, extended clear zone with low surface tension, and good BATH percentage are among the best biosurfactants producers. Depending on lowest surface tension and highest E_24_%, isolate Z47 showed high significance (*p* < 0.0001) as compared with others. Therefore, it was selected as a promising biosurfactant producer to be molecularly identified and employed in our study.

### 3.3. Identification of the Most Potent Bacterial Isolates

**Figure 4:**
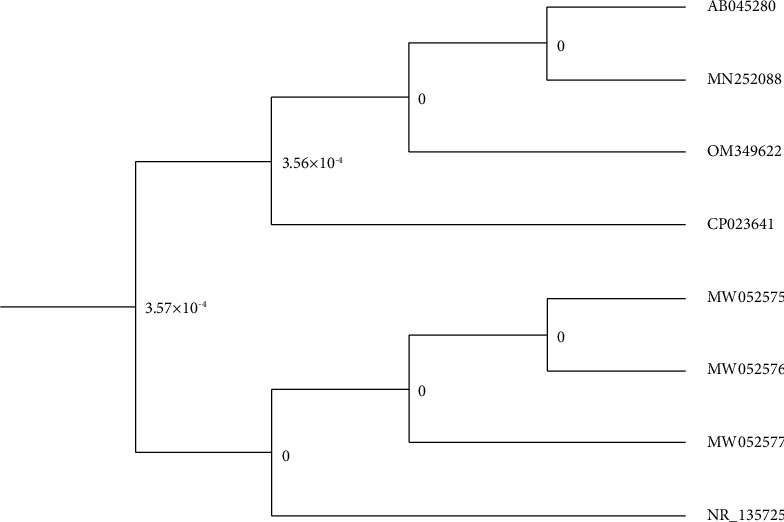
Phylogenic tree of *P. guguanensis* strain Iraqi ZG.K.M in NCBI database.

Based on the BLAST analysis in the NCBI and from the taxonomic analysis (Table 3) and phylogeny tree (Figure 4), it was revealed that the selected isolate (Z47) belongs to *Pseudomonas guguanensis*, which has 100% pairwise similarity with *Pseudomonas guguanensis* strain A52, isolated from petroleum-contaminated soil in China. The Novel bacterium was recorded in the NCBI GenBank nucleotide sequence database as *Pseudomonas guguanensis* strain Iraqi ZG.K.M with accession number OM349622.

### 3.4. Optimization of Biosurfactant Production by *Pseudomonas guguanensis* Strain Iraqi ZG.K.M

To get high productivity of biosurfactant by *P. guguanensis* strain Iraqi ZG.K.M, growth optimization was performed to determine the medium composition and optimum cultivation conditions, by changing one variable at a time with keeping other factors constant. Surface tension and E_24_% were measured as a response.

**Figure 5:**
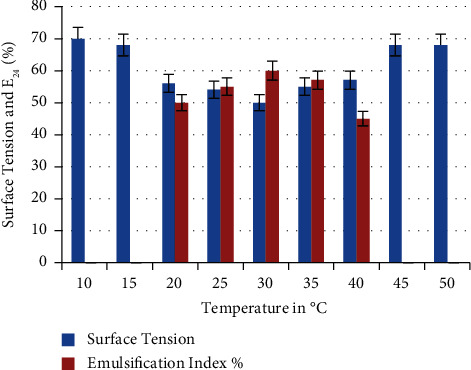
Effect of different incubation temperatures on biosurfactant production. 30°C was the optimum temperature with the highest E_24_% (60 ± 0.33%) and lowest surface tension at 50 ± 0.33 mN/m.

Results presented in Figure 5 showed the capability of *P. guguanensis* strain Iraqi ZG.K.M to grow and produce biosurfactant at a wide range of temperatures, including 20, 25, 30, 35, and 40°C. The optimum temperature for biosurfactant production was 30°C with the highest E_24_% (60 ± 0.33%) and lowest surface tension 50 ± 0.33 mN/m. The bacterium was unable to grow at 10, 15, 45, and 50°C. These results were in accordance with Ramya Devi and his colleagues who used 30°C as the optimum temperature for biosurfactant production by *P. guganensis* [27]. Pardhi and his colleagues found that 35 ± 2°C was the best temperature required for biosurfactant production from *P. guguanensis* D30 [28]. Temperature is the most important parameter affecting biosurfactant production, as maximum enzymatic activation can only be obtained at an optimum temperature [27].

**Figure 6:**
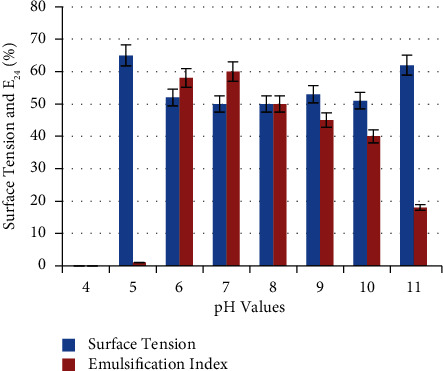
Effect of different pH values on biosurfactant production. pH 7 was the optimum with the highest E_24_% (60 ± 0.33%) and lowest surface tension 49 ± 0.33 mN/m.

From Figure 6, a good activity of bacterial growth was recorded with pH values (6–10), but the best production of biosurfactant was recorded at pH values (6–8), followed by decreasing in biosurfactant productivity. The highest E_24_% (60 ± 0.33%) and lowest surface tension (49 ± 0.33 mN/m) were recorded at pH 7. At pH 5, there was weak bacterial growth, while it was inhibited at pH 4. Therefore, components of biosurfactant may be precipitated at low pH values, which contributes to increasing the measurement of surface tension and decreasing the E_24_%.

A similar result was obtained for *P. guguanensis* D30 which produced the highest biosurfactant at pH 7 [28]. However, Fouda and his colleagues found that rhamnolipid production in *P. aeruginosa* 4.2 was recorded at a pH range of 7-8 [29].

**Figure 7:**
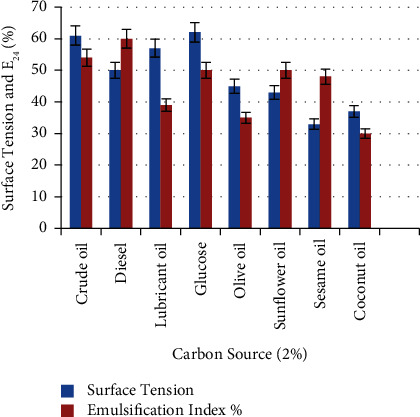
Effect of different carbon sources on biosurfactant production. Sesame oil is the best with E_24_% (48 ± 0.33%) and the lowest surface tension (33 ± 0.67 mN/m).

Several kinds of carbon sources have been investigated for biosurfactant production, as there is a correlation between microbial growth and biosurfactant production on hydrocarbons [28]. Results indicated in Figure 7 showed the ability of *P. guguanensis* strain Iraqi ZG.K.M to degrade a wide range of carbon sources (at 30°C and pH7) and produce biosurfactant. Sesame oil is the best used carbon source, as it provides a good level of emulsification (48 ± 0.33%) with the lowest surface tension (33 ± 0.67 mN/m) followed by sunflower oil with E_24_% (50 ± 0.33%) and surface tension (43 ± 0.33 mN/m). Diesel was also considered a good carbon source, allowing it to produce biosurfactant with a (60 ± 0.33%) emulsifying index and (43 ± 0.33 mN/m) surface tension. Pardhi and his colleagues demonstrated that biosurfactant production from *P. guguanensis* D30 was induced when using mineral oil as a carbon source [28]. Fouda and his colleagues found that *P. aeruginosa* 4.2 was easily utilizing glycerol as a carbon and energy source [29]. While Motwali and his colleagues indicated that olive oil was the best carbon source for *P. balearica*, providing the highest level of emulsification and cell dry weight [15].

**Figure 8:**
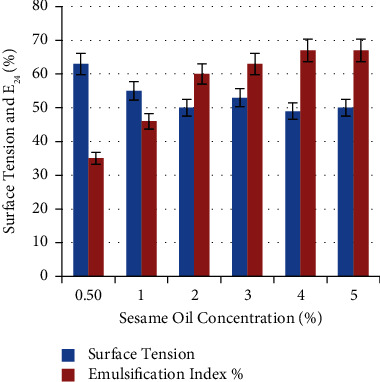
Effect of different sesame oil concentrations on biosurfactant production. 4% was the best concentration with better values of E_24_% (67 ± 0.33%) and surface tension 49 ± 0.67 mN/m.

Different concentrations of sesame oil were used to determine the optimal concentration for biosurfactant production by *P. guguanensis* strain Iraqi ZG.K.M. Results showed that the gradual increase in carbon source concentration was accompanied by an increase in emulsification index and decrease in surface tension (Figure 8). These dramatic changes in emulsifying activity and surface tension reached their better values of 67 ± 0.33% and 49 ± 0.67 mN/m at a concentration of 4%. These results were greatly similar to that reported by Pardhi and his colleagues [28]. They reported that hydrocarbon concentration plays an important role in *P. guguanensis* D30 for biosurfactant synthesis and indicated that the highest E_24_% (70%) was achieved with 8% mineral oil. Likewise, Tong and his colleagues used 5% crude oil to produce biosurfactant from *Pseudomonas* sp. BS1 [30].

**Figure 9:**
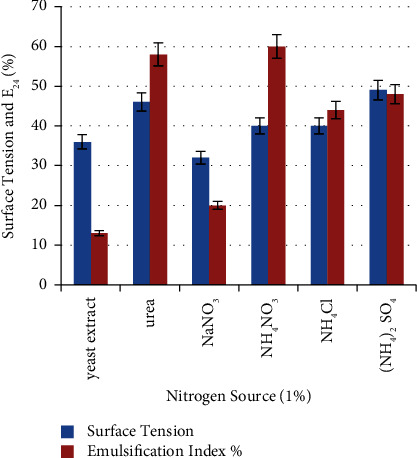
Effect of different nitrogen sources on biosurfactant production. NH_4_NO_3_ provides optimal results with a 60 ± 0.33% emulsification index and 49 mN/m surface tension.

Nitrogen source plays an important role in the production of biosurfactants since it is essential for microbial growth and enzyme production [28]. From Figure 9, it is clear that bacterial ability for biosurfactant production varies with different nitrogen sources. Ammonium nitrate (NH_4_NO_3_) provides optimal results in measured parameters, with a 60 ± 0.33% emulsification index and 49 mN/m surface tension. Urea was also suitable for biosurfactant production, with a 58 ± 0.33% emulsification index and 46 ± 0.33 mN/m surface tension. Motwali and his colleagues, Pardhi and his colleagues, and Fouda and his colleagues obtained similar results; they found that NH_4_NO_3_ is the best nitrogen source for biosurfactant production by *P. balearica*, *P. guguanensis* D30, and *P. aeruginosa*, respectively [15, 28, 29].

**Figure 10:**
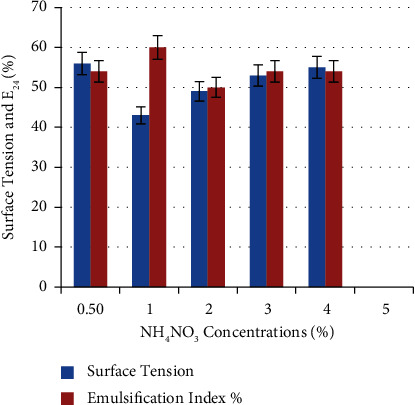
Effect of NH_4_NO_3_ concentrations on biosurfactant production. A maximum E_24_% (60 ± 0.33%) and minimum surface tension (49 mN/m) were obtained when NH4NO3 was added in a concentration of 1% (w/v).

Figure 10 shows the ability of *P. guguanensis* strain Iraqi ZG.K.M to produce biosurfactants in various NH_4_NO_3_ concentrations. A maximum E_24_% (60 ± 0.33%) and minimum surface tension (49 mN/m) were obtained when NH_4_NO_3_ was added in a concentration of 1% (w/v). When the concentration of NH_4_NO_3_ is above or below 1%, there was a reduction in E_24_% and an increase in surface tension. Similarly, Pardhi and his colleagues found that a BH medium containing 1% NH_4_NO_3_ may induce the production of biosurfactant by *Pseudomonas guguanensis* D30 [28]. Likewise, the best results for *P. aeruginosa* 4.2 and *B. cereus* 2.3 were obtained by Fouda and his colleagues, using 1% NH_4_NO_3_ [29]. Motwali and his colleagues used 1% NH_4_NO_3_ as the optimal concentration for biosurfactant production from *P. balearica* [15]. Nitrogen limitation was reported to increase the rhamnolipid production by *P. aeruginosa* PBSC1 [31].

**Figure 11:**
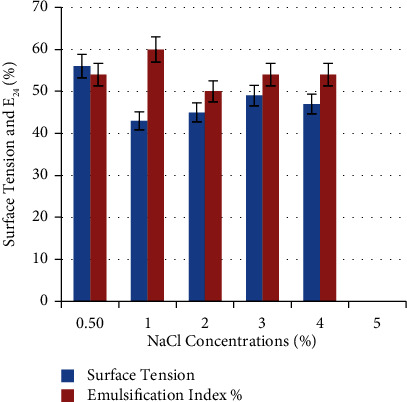
Effect of NaCl concentrations on biosurfactant production. A maximum E_24_% (54 ± 0.33%) and minimum surface tension (55 ± 0.33 mN/m) were obtained at 1% (w/v) NaCl.

NaCl concentration in certain media can influence biosurfactant production from microorganisms. Results illustrated in Figure 11 show the ability of the bacterial strain to tolerate various NaCl concentrations and produce biosurfactant. A maximum E_24_% (54 ± 0.33%) and minimum surface tension (55 ± 0.33 mN/m) were obtained when NaCl was added in a concentration of 1% (w/v). Fouda and his colleagues stated that culture media with 0.5% NaCl leads to a maximum production of biosurfactant by *B. cereus* 2.3 and *P. aeruginosa* 4.2, and the productivity directly decreased parallel to salt concentration until 8% [29]. Another study reported the ability of *P. aeruginosa* DHT2 to tolerate salinity up to 10% due to biosurfactant production. NaCl is known to activate the biosurfactant performance in bacterial strains isolated from oil reservoirs or seawater [32].

**Figure 12:**
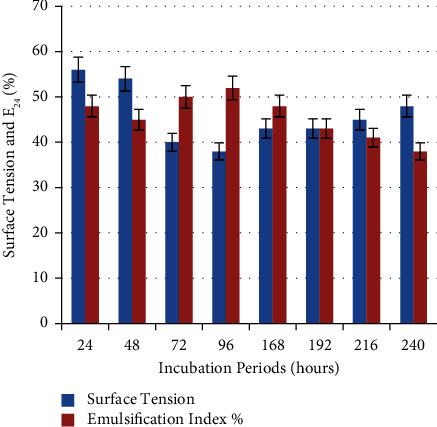
Effect of different incubation periods on biosurfactant production. 96 h was considered as the optimal incubation period for biosurfactant harvesting in *P. guguanensis* strain Iraqi ZG.K.M.

As shown in Figure 12, biosurfactant appeared at the same time as bacterial growth, and there is a gradual increase in biosurfactant production with increasing incubation period until it reached to maximum E_24_% (52 ± 0.33%) and lowest surface tension (38 ± 0.33 mN/m) at 96 h as compared with another incubation period. So, in *P. guguanensis* strain Iraqi ZG.K.M, 96 h was considered as the optimal incubation period for biosurfactant harvesting. This indicates that biosurfactant production occurs during the stationary phase and the product formation appeared to be partly growth-associated [33]. The E_24_% was decreased after 96 h of incubation and this may be attributed to the interference between metabolites and emulsion formation [34].

Pardhi and his colleagues reported that *P. guguanensis* D30 could produce the maximum amount of biosurfactant on the 5th day of shaking incubation at 35°C [28]. Fouda and his colleagues reported that the optimal incubation period for *B. cereus* 2.3 and *P. aeruginosa* 4-2 were (60–72) h and (48–72) h, respectively [29]. Also, Xia and his colleagues pointed out that rhamnolipids are typical secondary metabolites and their production was significantly increased in the stationary phase after 48 h, 132 h, and 120 h of fermentation in *P. aeruginosa* RS29, *P. fluorescence*, and *P. aeruginosa* 181, respectively [35]. While, Motwali and his colleagues reported that the best E_24_% and highest cell dry weight were recorded after 312 h of fermentation in *P. balearica* [15].

### 3.5. Production and Extraction of Biosurfactant

**Figure 13:**
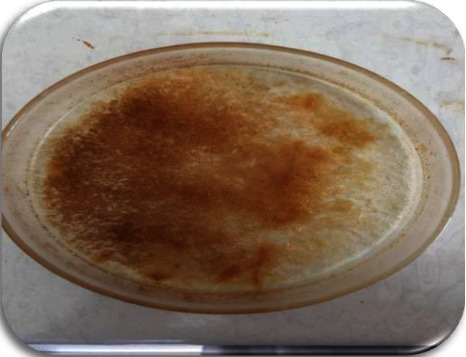
Partially purified biosurfactant using the acid precipitation and chloroform : methanol (2 : 1 v/v) solvent extraction method.

For biosurfactant extraction from *P. guguanensis* strain Iraqi ZG.K.M, results showed that chloroform : methanol was a good solvent as compared to ethyl acetate, where chloroform : methanol gave 3.01 g/l of a dark yellowish oily precipitate of biosurfactant (Figure 13), while ethyl acetate gave the least yield (1.13 g/l). Results were greatly matched with results reported by Adebajo and his colleagues [36]. They used acid precipitation and different solvents including ethyl acetate, dichloromethane, acetone, and chloroform : methanol (2 : 1 v/v) for biosurfactant extraction from *P. putida*. They found that chloroform : methanol gave the highest quantity of biosurfactant, followed by acetone and dichloromethane, and the least yield has been recorded with ethyl acetate. Patowary and his colleagues employed acid precipitation and ethyl acetate for biosurfactant extraction from *P. aeruginosa*, which resulted in 2.26 g/L honey brown crude biosurfactant [20]. While, Tripathi and his colleagues used acid precipitation and chloroform : methanol (2 : 1 v/v) to extract biosurfactants from *Microbacterium esteraromaticum* IITR47, *Ochrobactrum anthropi* IITR07, *Pseudomonas mendocina* IITR46, *P. aeruginosa* IITR48, and *Stenotrophomonas maltophilia* IITR87, which yielded 804, 981, 510, 360, and 1146 mg/l of biosurfactants, respectively [19]. On the other hand, Sumiardi and his colleagues obtained 3.24 g/l of biosurfactant, produced by *B. subtilis* strain ANSKLAB03, using the acid precipitation and chloroform : methanol (2 : 1 v/v) extraction method [2].

### 3.6. Characterization of Partially Purified Biosurfactant

#### 3.6.1. Determination of Ionic Nature

**Figure 14:**
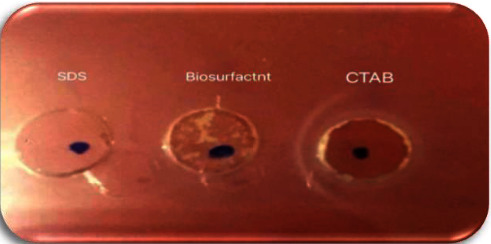
The agar double diffusion test showing the anionic nature of biosurfactant produced by *P. guguanensis* strain Iraqi ZG.K.M versus different tension-active agents of known charges.

The agar double diffusion test, based on passive diffusion of two substances having the same or opposite charges in a low degree of hardness medium, clarified the anionic nature of biosurfactant, in which a line of precipitation was formed between the cationic compound (CTAB) and the partially purified biosurfactant. While, no precipitation line was formed between biosurfactant and SDS (Figure 14). Ibrahim mentioned the anionic character of surfactants produced by *Citrobacter freundii* HM-2 and *Ochrobactrum anthropi* HM-1, using the same test [21].

#### 3.6.2. Thin-Layer Chromatography (TLC) Analysis

**Figure 15:**
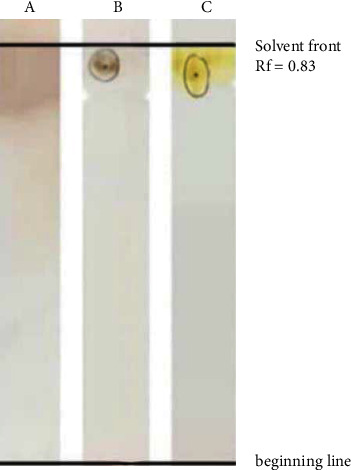
TLC analysis of partially purified biosurfactant extracted from *P. guguanensis* strain Iraqi ZG.K.M. The chromatograms were (A) sprayed with 1% ninhydrin solution, (B) sprayed with Molish's reagent, and (C) exposed to iodine vapor.

TLC analysis was conducted for preliminary characterization of the partially purified biosurfactant. Results showed a single definite spot on the TLC plates, with an *R*_*f*_ value of 0.83 (Figure 15). This spot gave positive results for lipids and sugars, detected as yellow spot and brown colored spot when exposed to iodine vapor and Molisch's reagent, respectively. In contrast, a negative reaction was observed towards the ninhydrin solution, which indicates the absence of amino groups. This suggested the glycolipid nature of this biosurfactant and could be considered a monorhamnolipid as compared with biosurfactants produced from *P. aeruginosa*. Sun and his colleagues revealed the composition of glycolipid produced from *P. aeruginosa* CQ2 by TLC analysis, which formed two spots of mono and dirhamnolipid with *R*_*f*_ values 0.85 and 0.53, respectively [17].

#### 3.6.3. Fourier Transform Infra-Red (FT-IR) Analysis

**Figure 16:**
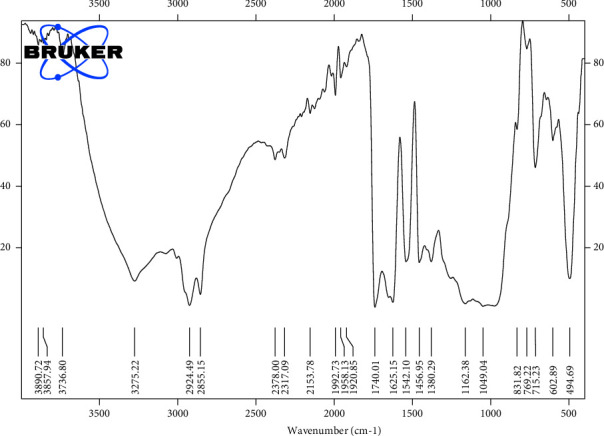
FTIR spectrum of partially purified biosurfactant produced by *P. guguanensis* strain Iraqi ZG.K.M.

The partially purified biosurfactant showed clean FT-IR vibration peaks, which support the proposed structure (Figure 16). The spectral data from the partially purified biosurfactant clarify the presence of the alcoholic hydroxyl (−OH) group as a sharp peak in the region 3275 cm⁻^1^, indicating the involvement of hydroxyl groups in intramolecular hydrogen bonding. The absorption bands at 2924 cm⁻^1^ and 2855 cm⁻^1^ were attributed to the characteristic stretching mode of long acyl chains of the -CH_2−_ and -CH_3−_ groups. The stretching vibrations revealed at 1740 cm⁻^1^ and 1625 cm⁻^1^ confirm the presence of aliphatic ester carbonyl C=O and C-O groups, respectively. Stretching vibrations revealed at 1456 cm⁻^1^ and 1380 cm⁻^1^ indicate the presence of alkyl groups that demonstrate the presence of bonds between hydroxyl groups and the carbon atoms in the chemical structure of the glycoside portion [17, 22]. It was revealed that the broad peck region at 1162 cm⁻^1^ and 1049 cm⁻^1^ are associated with (C–O–C) stretching vibrations of polysaccharides [37]. Finally, characteristic peaks appearing between 715 cm⁻^1^ and 494 cm⁻^1^ were marked as anomeric carbon of the carbohydrate fingerprint [38]. Compared to previous studies, all these main chemical groups were in line with the structural characteristics of rhamnolipid, but it needs further characterization. The results of IR spectra were highly similar to that of glycolipids produced by *P. aeruginosa* strains [17, 19, 21]. Jha and his colleagues revealed the composition of cyclic lipopeptides-surfactin and fengycin, produced by *B. subtilis* R1 strain, using the FT-IR spectrum [39]. While, Sharma and his colleagues used the FT-IR spectrum to reveal the composition of biosurfactant produced by *Enterococcus faecium*, which was lipid and polysaccharide fractions [25].

#### 3.6.4. Gas Chromatography-Mass Spectrometry (GC-MS) Analysis

**Figure 17:**
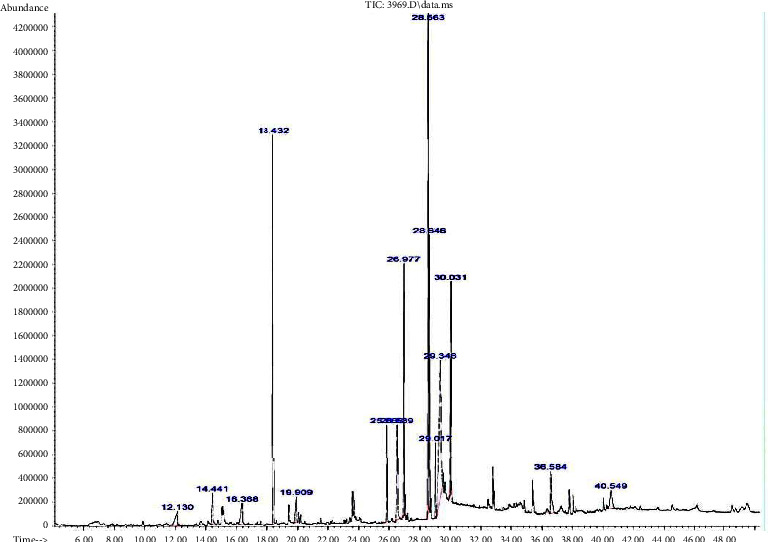
GC-MS analysis of the partially purified biosurfactant produced by *P. guguanensis* strain Iraqi ZG.K.M. Methyl linoleate was the predominant fatty acid which gave a predominant peak at RT 28.56 min.

With the use of GC-MS, the number, chemical composition, and molecular weights of components present in the partially purified biosurfactant were revealed. Results illustrated in Table 4 show nineteen different components present in the extracted biosurfactant, and they were separated at different retention times (RT) ranging from 12.129 to 40.547 min. All these compounds comprised of long chain fatty acids, mainly C-16 long fatty acids [25]. The fatty acids n-hexadecanoic acid, ethyl palmitate, methyl linoleate, 8-octadecenoic acid, methyl ester, methyl stearate, ethyl stearate, methyl palmitate, dodecanoic acid, linoleic acid, 2,4-di-tert-butylphenol, and 9,17-octadecadienal are the major fatty acids that showed prominent peaks (Figure 17). The predominant fatty acid was methyl linoleate which gave a predominant peak at RT 28.56 min. Therefore, results indicate the rhamnolipid nature of the biosurfactant and usually to be monorhamnolipid.

Ramya Devi and his colleagues mentioned the chemical nature of emulsifiers, produced from *P. guguanensis* by GC-MS [27]. They isolated an unusual, high molecular weight monorhamnolipid which is attached to esters of palmitic and stearic acids. GC-MS data revealed the rhamnolipid nature of the biosurfactant produced by the *P. aeruginosa* SMVIT1 strain [40]. While, GC-MS data of Thio and his colleagues recorded the presence of rhamnolipid precursors in the form of *β*-hydroxy fatty acids, thus confirming the rhamnolipid nature of biosurfactant produced from *Pseudomonas* sp. LM19 [23]. Sharma and his colleagues found from the GC-MS data that the fatty acid produced by *E. faecium* was hexadecanoic acid [25].

## 4. Conclusions

In conclusion of the current study, contaminated soil with hydrocarbons was considered a good source for the isolation of oil-degrading bacteria which can then be used for bioremediation. These bacteria have a good ability to live and utilize crude oil and hydrocarbon residues, due to their ability to produce different amounts of biosurfactants as compared with each other. Biosurfactants are organic microbial products that proved as good replacements for synthetic surfactants because of their biodegradability, low toxicity, and environmentally friendly nature. *Pseudomonas guguanensis* strain Iraqi ZG.K.M, isolated for the first time in Iraq from hydrocarbons contaminated soil, was one of the most efficient biosurfactant producers, assured by all screening tests. Furthermore, the nutritional factors and growth conditions have significant effects on the productivity of the biosurfactant. Therefore, optimization of growth conditions, MSM medium (pH 7) containing sesame oil (4%), NH₄NO_3_ (1%), and NaCl (1%) and incubated in shaking condition at 35°C for 96 hrs can maximize biosurfactant production which makes this bacteria competent enough. Extracted partially purified biosurfactants possess high surface activity (38 ± 0.33 mN/m) and excellent emulsification activities (52 ± 0.33%). It was characterized as a rhamnolipid with anionic nature and usually to be monorhamnolipid. Since there are no reports of *P. guguanensis* pathogenicity, monorhamnolipids can be seen as a viable solution for large-scale production as commercial eco-friendly hydrocarbon dispersants.

## Figures and Tables

**Table 1:** Factors considered for optimization of biosurfactant production from identified strain.

Factor	Range
Incubation temperature	10, 15, 20, 25, 30, 35, 40, 45, and 50°C
pH	4, 5, 6, 7, 8, 9, 10, and 11
Carbon source	Crude oil, diesel, engine oil, glucose, olive oil, sunflower oil, sesame oil, and coconut oil
Carbon source concentration	0.5, 1, 2, 3, 4, and 5% (w/v)
Nitrogen sources	(NH_4_)_2_ SO_4_, NaNO_3_, NH_4_NO_3_, NH_4_Cl, yeast extract, and urea
Nitrogen sources concentration	0.5, 1, 2, 3, and 4% (w/v)
Salt concentration	0.5, 1, 2, 3, and 4% (w/v)
Incubation period	24, 48, 72, 96, 168, 192, 216, 240, and 264 h

**Table 2:** Secondary screening of bacterial isolates for biosurfactant production.

No.	Isolate symbol	Drop collapse assay	Oil spreading assay (mm)	Surface tension	Emulsification index E_24_ (%)	BATH assay (%)
1	Z 4	+	—	62.67 ± 0.33	13 ± 1	20.33 ± 0.33
2	Z 5	+	48.33 ± 1.76	52	49 ± 1	66.33 ± 0.88
3	Z 7	+	33.33 ± 1.66	48.67 ± 0.67	43.66 ± 0.33	58.33 ± 0.88
4	Z 9	+	—	69.33 ± 0.33	—	—
5	Z 11	+	10.66 ± 0.66	67	—	12.66 ± 0.88
6	Z 14	+	7.66 ± 0.88	60.33 ± 0.33	—	15.33 ± 1.20
7	Z 25	+	30.33 ± 1.76	59	50.33 ± 0.33	74 ± 2.08
8	Z 31	+	77.66 ± 2.96	49	61	76.33 ± 0.88
9	Z 33	+	44.33 ± 0.33	52 ± 1	49.66 ± 1.45	64.33 ± 1.85
10	Z 34	+	—	68.33 ± 0.33	—	—
11	Z 35	+	23.33 ± 2.18	59	16.66 ± 0.33	30.33 ± 1.33
12	Z 36	+	10 ± 1.52	62.67 ± 0.33	10.33 ± 0.88	18.66 ± 1.76
13	Z 40	+	21.66 ± 0.33	61.67 ± 0.33	15	29.66 ± 0.88
14	Z 43	+	26.33 ± 0.33	60.67 ± 0.33	22.66 ± 0.33	56.66 ± 0.33
15	Z 44	+	—	61.33 ± 0.33	—	9.66 ± 0.66
16	**Z 47**	+	**77.66** **±** **0.33**	**44**	**64.66** **±** **0.33**	**88.33** **±** **2.18**
17	Z 48	+	73.33 ± 1.20	49	50.66 ± 0.66	71.33 ± 0.88
18	Z 49	+	39.33 ± 0.33	61	47.33 ± 0.33	56.33 ± 1.20
19	Z 50	+	9.33 ± 1.66	59 ± 1.73	32.66 ± 1.20	57 ± 1
20	Z 51	+	3.66 ± 1.33	69	—	9.66 ± 1.20
21	Z 52	+	12.66 ± 1.20	67	9.66 ± 0.66	10.66 ± 0.33
22	Z 53	+	27.33 ± 1.85	64.67 ± 0.33	22.66 ± 0.88	11.66 ± 1.33
23	Z 54	+	48.33 ± 0.33	56.33 ± 0.33	44.33 ± 0.33	65.66 ± 1.85
24	Z 56	+	39 ± 1.52	61	48.66 ± 0.33	58.66 ± 0.33
25	Z 60	+	36.33 ± 0.33	59.33 ± 0.67	26.66 ± 0.33	51.33 ± 0.33
26	Z 66	+	—	65	—	14.33 ± 0.66
27	Z 71	+	38 ± 2.08	55.33 ± 0.67	37.66 ± 0.33	5.66 ± 0.66
28	Z 77	+	49.33 ± 0.33	50.33 ± 0.33	36.33 ± 0.66	61.66 ± 2.02
29	Z 80	+	22 ± 2.08	59	10.66 ± 0.33	30.66 ± 1.20

Data: mean ± SEM. The experiments were performed in three replicates. The bold values indicate that Z47 showed the higher E24% and the lowest surface tension, so, it is the best biosurfactant producer.

**Table 3:** Taxonomic analysis of *P. guguanensis* strain Iraqi ZG.K.M in NCBI database. It has 100% pairwise similarity with *P. guguanensis* strain A52.

Description	Mix score	Total score	Query cover (%)	E value	Per. ident	Accession
*Pseudomonas guguanensis* strain A52 16S ribosomal RNA gene, partial sequence	2593	2593	100	0.0	100.00%	MN252088.1
*Pseudomonas guguanensis* strain 4-B1 16S ribosomal RNA gene, partial sequence	2582	2582	100	0.0	99.86%	MW052575.1
*Pseudomonas guguanensis* strain CC-G9A 16S ribosomal RNA gene, partial sequence	2579	2579	100	0.0	99.79%	NR 135725.1
*Pseudomonas guguanensis* strain 4-B3 16S ribosomal RNA gene, partial sequence	2573	2573	99	0.0	99.93%	MW052577.1
*Pseudomonas guguanensis* strain 4-B2 16S ribosomal RNA gene, partial sequence	2569	2569	99	0.0	99.93%	MW052576.1
*Pseudomonas guguanensis* 16S ribosomal RNA gene, partial sequence	2553	2553	98	0.0	99.93%	KU302611.1
*Pseudomonas guguanensis* strain PPS-12 16S ribosomal RNA gene, partial sequence	2521	2521	100	0.0	99.00	MT588845.1
*Pseudomonas guguanensis* strain FX03 16S ribosomal RNA gene, partial sequence	2514	2514	100	0.0	99.00%	KX585259.1
*Pseudomonas guguanensis* strain 4-n-1 16S ribosomal RNA gene, partial sequence	2507	2507	100	0.0	98.86%	MT127788.1
*Pseudomonas guguanensis* strain S7 16S ribosomal RNA gene, partial sequence	2501	2501	100	0.0	98.79%	MK89555.1
*Pseudomonas guguanensis* strain GS165 16S ribosomal RNA gene, partial sequence	2473	2473	99	0.0	98.50%	MN818648.1
*Pseudomonas guguanensis* strain KNDSS-Mac2 16S ribosomal RNA gene, partial sequence	2462	2462	100	0.0	98.29%	KY471631.1

**Table 4:** The chemical composition of the partially purified biosurfactant produced by *P. guguanensis* strain Iraqi ZG.K.M and analyzed by GC-MS.

No.	RT (min)	Area%	Name	Quality
1	12.129	1.93	2-Octenoic acid	90
2	14.443	2.02	2H-Pyran-3,4,5-triol and tetrahydro-2-methoxy-6-methyl-	64
3	15.087	1.38	Methyl.alpha.-L-fucopyranoside	64
4	15.164	0.78	2H-Pyran-3,4,5-triol and tetrahydro-2-methoxy-6-methyl-	80
5	16.368	1.94	2-Decenoic acid	64
6	18.433	11.74	2,4-Di-tert-butylphenol	95
7	19.43	1.09	Dodecanoic acid	98
8	19.907	1.86	3-Isopropyl-5-methyl-hex-4-en-2-one	41
9	25.863	2.97	Methyl palmitate	98
10	26.538	5.75	*n*-Hexadecanoic acid	99
11	26.979	8.22	Ethyl palmitate	99
12	28.562	16.83	Methyl linoleate	99
13	28.645	7.54	8-Octadecenoic acid and methyl ester	99
14	29.018	2.22	Methyl stearate	99
15	29.345	18.70	Linoleic acid	97
16	30.03	6.72	Ethyl stearate	99
17	32.801	2.34	1-Nonadecene	90
18	36.583	3.43	9,17-Octadecadienal, (Z)-	95
19	40.547	2.53	4-Carbomethoxy-6,7-dimethoxy-3(3′,4′-dimethoxyphenyl)-isoquinoline	52

## Data Availability

The data used to support the findings of this study are available from the corresponding author upon request.
